# Progress on the Origin of Species

**DOI:** 10.1371/journal.pbio.0030062

**Published:** 2005-02-15

**Authors:** Douglas J Futuyma

## Abstract

Two new books on speciation update the classic texts by Mayr and Grant and help set the stage for a renaissance of research into one of the most important processes in evolution

Despite the biases that a single author inevitably brings to a subject, only one or a few closely interacting authors can bring coherence, synthesis, and vision to a broad and complex topic. A symposium volume just doesn't do the job. Few topics in biology are as simultaneously encompassing, complex, and controversial as the origin of species, i.e., speciation. Speciation is, after all, the process responsible for biological diversity, at least of sexual organisms, so it is hardly a minor topic. But even though recent years have seen the publication of symposia on speciation and books on the ever-contentious issue of species concepts, it has been 23 years since Verne Grant's authoritative *Plant Speciation* [1] and 41 years since Ernst Mayr's magisterial and highly influential *Animal Species and Evolution* [2]—the last syntheses of research on speciation. Now, two outstanding new books not only treat speciation as a conceptually unified topic in both plants and animals for the first time, but also provide rich review and analysis of a vast subject that has progressed at least as much since Mayr and Grant wrote as in the century that preceded their work.

These books are very different, but wonderfully complementary. Gavrilets reviews and adds to mathematical theories and simulation studies of speciation and related issues, such as fitness landscapes and selection in heterogeneous environments. A deep reading of his book will require considerably more mathematical competence than most evolutionary biologists (including this reviewer) have, but Gavrilets provides excellent verbal explanations of the models' assumptions and conclusions, as well as comparisons and critiques of related models. Gavrilets cites empirical studies (with which he has very broad familiarity) plentifully, but as a theoretician, he does not evaluate them or describe them in depth. That task is undertaken by Coyne and Orr, who introduce most topics with a verbal overview of theory, review empirical evidence and its bearing on hypotheses, and conclude with incisive assessments of what they think we know and what remains uncertain or unexplored. Like Gavrilets, they offer a number of novel ideas or suggestions about how to proceed. Coyne and Orr have both worked mostly on speciation genetics in Drosophila, so it is hardly surprising that their treatment of speciation bears a strong genetic emphasis and draws heavily on Drosophila work (perforce, since this is almost the only source of evidence on some topics, such as the genetics of hybrid sterility and inviability). Even the most drosophilophobic readers, however, will be pleased by the extent to which Coyne and Orr have conscientiously scoured the literature on nonmodel animals and plants.

To appreciate the landmark status of these books, consider what has happened in speciation studies since Mayr and Grant published theirs. Mayr and Grant articulated positions on species and speciation that had developed during and soon after the Modern Synthesis of the 1930s and 1940s, when modern evolutionary theory developed from a reconciliation of genetics, systematics, and paleontology. Mayr and Grant drew on abundant systematic data on patterns of divergence and experimental data on genetic differences between related species. They rightly identified reproductive isolation (RI) as a critical, even defining, property of species, and allopatric divergence (i.e., in disjunct geographic areas) as the major geographic mode of speciation. They recognized that trait differences between species, including RI, usually have a polygenic basis, and that different coadapted (epistatically interacting) sets of genes underlie incompatibility (e.g., hybrid sterility). They emphasized the role of ecological selection as a driving force in speciation, largely by extrapolation from the primacy of selectionist thinking that developed during the Synthesis. They accepted that natural selection can reinforce prezygotic isolation (i.e., lack of mating or zygote formation) between species and thereby reduce production of unfit hybrids, even if Mayr did not share Dobzhansky's belief that this was the norm. Mayr combined selection with genetic drift in his theory of founder-effect speciation (divergence in populations founded by just a few individuals), which became widely accepted. (It became Eldredge and Gould's [3] theoretical foundation for punctuated equilibrium nine years after Mayr's book appeared.) Mayr and Grant wrote against a background that almost entirely lacked any mathematical theory of speciation (which I suspect neither of them would have drawn on even if it had been developed), any relevant molecular data (other than early allozyme studies by the time Grant published), and any rigorous phylogenetic methodology. Kimura's neutral theory of molecular evolution [4] had not yet been published when Mayr wrote, and had not been vindicated when Grant wrote, so genetic drift and a neutral (nonselectionist) interpretation of molecular data were still suspect. Detailed analysis of genetic architecture was decades away, and of course insights into selection and historical demography from DNA sequence data were a dim dream at best.

Coyne and Orr and Gavrilets analyze a new world of speciation studies. Theoretical studies of speciation, for example, now include more than 100 papers on one topic alone, the evolution of prezygotic isolation. (Gavrilets laments that the theoretical work suffers from domination by simulation rather than analysis, so that it is often hard to draw general conclusions from models that use different assumptions, but he nevertheless draws some fairly strong conclusions, as I note below.) Molecular studies have provided important data on such issues as the absolute dates of speciation events, the duration of speciation, and the time course of the evolution of RI. The field of molecular phylogeography, which documents the history of spatial isolation and geographic expansion of populations, has developed. The relation between range overlap of related species and their molecularly dated time of divergence provides some evidence on the role of geographic versus sympatric speciation (i.e., speciation without geographic segregation). In all these areas and others, our knowledge has increased steadily. For instance, molecular markers enable more detailed dissection of the genetic architecture of species differences, and support the conclusion that they are usually rather highly polygenic, but that much of the variance can be explained by a few major gene substitutions. We now have good evidence, as Coyne and Orr emphasize, that at least in animals, hybrid infertility is caused by differences in gene action, not by structural chromosome differences or failure of meiosis.

Regarding the mechanisms of speciation, evidence for the role of divergent ecological selection in allopatric speciation is sparse, because this crucial topic has been unaccountably neglected until recently. Very different kinds of data, ranging from DNA sequences to correspondence between RI and ecological divergence, support natural selection, but there is hardly enough evidence, in my opinion, to support Coyne and Orr's strong conclusion that “at least one important debate has been settled: selection plays a much larger role in speciation than does drift” (p. 410). Even more astonishing than the paucity of studies of the role of ecological selection in speciation is the fact that the likely role of sexual selection was not even recognized until almost 20 years after Mayr's book. I agree with Coyne and Orr that the theory and evidence for speciation by sexual selection is one of the most important advances in speciation studies, but it is important to recognize that the evidence consists mostly of correlations between diversification rates and indices of the likely strength of sexual selection; as Coyne and Orr note, there are no cases in which we understand just how sexual selection has caused speciation. This is a rich, largely unexplored area. Gavrilets feels that divergent evolution by sexual conflict (in which females evolve resistance to males' advances) is a potentially important process, whereas Coyne and Orr are skeptical that this will prove widespread. Coyne and Orr remark that populations may diverge in male signals because of intrasexual selection (competition among males), and that female mate preference may follow. Quite so, but even though Berglund et al. [5] summarized many examples in which male signals appear to serve both inter- and intrasexual functions, this topic has been almost ignored in the literature of both speciation and sexual selection. On a related theme, an important speciation process appears to be the extraordinarily rapid evolution of male reproductive proteins (e.g., sperm surface proteins), which may contribute to the “faster male evolution” that is a cause of “Haldane's rule” (that hybrid sterility and inviability first appear in the sex that has two unlike sex chromosomes).

Despite their very different approaches, Coyne and Orr and Gavrilets arrive at rather similar conclusions on some of the most controversial issues in speciation. One such is the role of genetic drift in speciation. Gavrilets analyzes founder-effect speciation (which combines drift and selection), agrees with most other theoreticians (e.g., Barton and Charlesworth) [6] that it is very improbable, and argues instead for his model of evolution on “holey landscapes,” whereby allopatric populations can evolve by genetic drift along ridges of roughly equal fitness to different, incompatible gene constitutions. He admits that the time to speciation under this process will ordinarily be very long unless selection is involved. Coyne and Orr fully accept both Barton and Charlesworth's critique and Gavrilets's alternative model. But while admitting the plausibility of Gavrilets's models of speciation by genetic drift, they nevertheless maintain that “the models seem unnecessary when compared to adaptive ones” (p. 398).

Coyne and Orr appear to adopt selection as the null hypothesis for speciation, whereas drift is generally taken as the null hypothesis in much of evolutionary genetics, for the simple reason that drift operates at all loci in all finite (i.e., real) populations, whereas selection need not. The burden of demonstrating that selection is *not* responsible for an evolutionary event (i.e., demonstrating a negative) is, of course, far heavier than the burden of demonstrating selection; indeed, Coyne and Orr do not address the difficult question of what would constitute evidence for drift. Having, perhaps, stacked the deck, Coyne and Orr find almost no evidence that drift has contributed to speciation in nature, but conclude that there is “considerable evidence” that selection has done so. However, the amount of evidence is hardly on a par with, say, the evidence for allopatric speciation. It consists of only about eight studies of ecological selection, indications that diversification rates are associated with greater scope for sexual selection, selection signatures in a few genes that underlie genetic incompatibility, and a paucity of molecular evidence for bottlenecks (i.e., opportunities for founder events) in the history of recently formed species. But the evidence on the role of sexual selection is very indirect, and the high levels of genetic variation revealed in molecular studies argue against past bottlenecks only if this is ancestral variation, rather than variation generated anew since a possible bottleneck—a question that has been addressed in only a few cases. Assuming that experiments with laboratory populations can be validly extrapolated to natural speciation processes, founder-effect speciation may indeed be a moribund hypothesis, but I do not believe long-term genetic drift can yet be ruled out, and cannot agree that this “important debate has been settled” (p. 410).

The geography of speciation continues to be one of the most difficult and contentious topics, and undoubtedly will remain so despite the careful analyses by these authors. They agree that parapatric speciation (evolution of RI between neighboring populations that exchange genes) is theoretically plausible, but Gavrilets notes that although it has become clear that its likelihood is sensitive to several model parameters, parapatric speciation is difficult to model and has been shamefully neglected. Coyne and Orr do not doubt that it is a fairly common mode of speciation, yet “it is almost impossible to demonstrate parapatric speciation in nature” (p. 118), and no cases have been well documented.

Gavrilets provides an exhaustive analysis of the many models of sympatric speciation, and identifies some key issues that have been underemphasized. For example, the sympatric evolution of behavioral isolation by “matching traits” (e.g., genetically independent male signal and female preference) is generally much more difficult than “similaritybased” mating (in which females choose males that have the same phenotypic trait as themselves). Just how common the latter is in animals is an open question that Coyne and Orr unfortunately do not address. Gavrilets also identifies the cost of female choosiness as a critical issue: many models of sympatric speciation depend on the assumption that females always succeed in mating even if the male type they prefer is rare, so their choosiness has no cost. Gavrilets criticizes some popular models of sympatric speciation on these and other grounds, and while granting that sympatric speciation by divergent habitat or host preference is plausible, he concludes that it need not be faster than allopatric speciation and that “contrary to common claims in recent theoretical papers, conditions for sympatric speciation are not wide and sympatric speciation does not occur easily” (p. 404).

For their part, Coyne and Orr feel that the prevalence of sympatric speciation is an empirical issue (but a very difficult one), and undertake a broad, detailed review. They identify three examples of completed speciation in which a sympatric scenario “seems plausible.” I see no reason to accept one of these cases, a pair of sister species of “parasites” (fig wasps) on the same host species, since allopatric speciation of a widespread parasite need not be accompanied by speciation of its host. Moreover, Coyne and Orr note weaknesses in all three cases, as well as in examples of “host races” that have been advanced as species *in statu nascendi*. Coyne and Orr's conclusion echoes Gavrilets's: “It is hard to see how the data at hand can justify the current wave of enthusiasm for sympatric speciation” (p. 178). *Bravi!*

I have indicated some disagreements with Coyne and Orr, and could certainly cite others. But whatever weaknesses their book may have (more ecology and phylogeny, anyone?) are much less important than its strengths. The strengths of *Speciation* are not only Coyne and Orr's comprehensive, scholarly coverage of an exceedingly broad subject, but also, and especially, their rigorous, incisive analysis, coupled with strongly stated conclusions and suggestions for how to resolve controversies. Many readers will have a visceral reaction against their position on sympatric speciation, reinforcement, founder-effect speciation, or other issues—but can these readers counter Coyne and Orr's arguments with equally cogent analysis? Or are these subjects that simply require more, and perhaps more imaginative, research?

Together, these books provide a comprehensive, thoughtful synthesis of our current understanding of one of the most important processes in evolution. They are required reading for anyone who studies species and speciation. I recommend *Speciation* and the nonmathematical final chapter (“General Conclusions”) of *Fitness Landscapes and the Origin of Species* to all evolutionary biologists, students, and professionals alike. It may not take another two decades for the next foundational books on speciation to appear, but these books will fill that role for a long time to come.

## Abbreviation

RI: reproductive isolation
